# Investigating factors associated to dysphagia and need for percutaneous endoscopic gastrostomy in patients with head and neck cancer receiving radiation therapy

**DOI:** 10.7150/jca.69130

**Published:** 2022-02-28

**Authors:** Petros Alexidis, Petros Bangeas, Konstantinos Efthymiadis, Konstantinos Drevelegkas, Pavlos Kolias

**Affiliations:** 1 Department of Radiation Oncology, Papageorgiou Hospital, Thessaloniki, Greece; 2 Aristotle University of Thessaloniki, 1 st University Surgery Department, Papageorgiou Hospital, Thessaloniki, Greece; 3 Medical Oncology Department, Papageorgiou Hospital, Aristotle University of Thessaloniki, Greece; 4 Diagnostic Medical Center, Thessaloniki, Greece; 5 Section of Statistics and Operational Research, Department of Mathematics, Aristotle University of Thessaloniki, Greece

**Keywords:** toxicity, radiotherapy, dysphagia, cancer

## Abstract

**Purpose:** In this study we sought to investigate factors associated to dysphagia and subsequent need for percutaneous gastrostomy (PEG) usage, in patients with head and neck cancer receiving radiation therapy.

**Methods:** The records of 123 patients with non-metastatic, stage I-IV head and neck cancer who were submitted to radiation therapy were retrospectively reviewed. Logistic regression models were used to investigate for associations between the outcomes of interest (grade ≥2 dysphagia and need for [PEG] usage) and potential predictive factors.

**Results:** Mean dose to pharyngeal constrictor muscles (OR=1.08, p=.002), concurrent chemotherapy (OR=3.78, p=0.015) and upper aerodigestive tract malignancies (OR=3.27, p=0.044) were associated with dysphagia grade≥2. A threshold of constrictors mean dose for dysphagia manifestation was also identified at 43 Gy (OR=4.51, p=0.002). Need for PEG use was correlated with definitive treatment (OR=7.03, p=.022), nasopharyngeal (OR=12.62, p=0.003), upper aerodigestive tract (OR=9.12, p=0.007) or occult primary malignancies (OR=10.78, p=0.016).

**Conclusion:** Patients suffering from upper aerodigestive tract malignancies, those with calculated constrictors mean dose >43 Gy, or planned to receive concurrent chemotherapy-radiotherapy should be closely monitored during treatment for dysphagia manifestation. Prophylactic PEG could be considered for patients receiving definitive therapy of the nasopharynx, upper aerodigestive tract or occult primary malignancies.

## Introduction

Head and neck cancer (HNC) is a common disease accounting for almost 4% of total cancer cases, while the new cases worldwide are estimated at 650,000 yearly^1^. Management of HNC varies according to disease stage and location of tumor. Treatment options include surgery, radiation therapy and chemotherapy which can be administered either as monotherapies or a combination. Radiation therapy plays an important role in the local management of HNC in both the definitive and postoperative setting. Most importantly, it allows for an organ preservation strategy by avoiding surgery, thus preserving organ function (voice, swallowing)^2^,^3^. Despite the favorable results of RT, treatment related toxicity has historically been a great challenge for radiation oncologists since it significantly affects quality of life and, in some cases, even compromises treatment effectiveness and patient's overall health status. Adverse events are still observed during treatment course, despite the technological advances in radiation oncology.

Among RT side effects, dysphagia is particularly important because it frequently affects patient's feeding ability leading to weight loss, dehydration, weakening of the immune system, health complications and subsequent treatment deintensification or interruptions. Excessive weight loss during treatment is a negative prognostic factor which has a significant impact on disease control^4^-^9^. Foreknowledge of factors that are associated with excessive dysphagia could help the treating physician individualize care during treatment or guide precautionary measures like prophylactic percutaneous endoscopic gastrostomy (PEG) placement and nutritional support.

## Materials and Methods

### Patients and interventions

This is an observational retrospective cohort study, investigating for factors related to dysphagia manifestation and need for PEG in patients with head and neck cancer receiving radiation therapy. Patients included were 18-90 years old and presented with non-metastatic, stage I-IV squamous cell carcinoma of the head and neck originating from any HN primary site. All patients were submitted to intensity modulated radiation therapy (IMRT) with or without concurrent chemotherapy and treatment was targeted to the primary site with optional irradiation of the neck lymph nodes according to indications. Both definitive and postoperative (adjuvant) treatments were allowed. Treatment was delivered to all patients with a schedule of 2 Gy/fraction and 5 fractions/week. Exclusion criteria were, alternating fractionation schedules (hyper or hypofractionation), metastatic disease, palliative treatment, previously irradiated patients, in field recurrence after prior RT, patients that have been treated for another HNC primary in the past, postoperative RT treatment due to recurrence, 3D conformal radiation therapy (3DCRT technique) and primaries not involving the swallowing route and not requiring lymph node irradiation.

The study's protocol was approved by the review board of General Hospital Papageorgiou of Thessaloniki. Due to the retrospective nature of the study, the need for patient informed consent was waved.

### Outcomes

The study's primary outcome was dysphagia manifestation which was defined as any event occurring during treatment or within 3 months after the end of it and was evaluated according to the Radiation Therapy Oncology Group (RTOG) scoring scale for toxicity^10^. The highest toxicity grade was recorded for each patient according to dysphagia severity and results were grouped to grade <2 and grade ≥2. The secondary outcome was need for PEG and was defined as need for use of PEG in those patients that was placed before treatment initiation or placement of PEG during treatment.

### Sample size calculation

The sample size was calculated with the intent to detect the effect of adding chemotherapy to RT on the primary outcome of interest, by adjusting for the confounders anatomical site, therapy type, treatment of the neck, type of neck irradiation and mean dose to the constrictor muscles. The expected rate of dysphagia grade ≥2 was 40% and the minimum effect size that the current study aimed to detect was 3. By setting the level of statistical significance at 0.05 and the power of the study at 0.8, the final sample size was calculated to 116 patients.

### Statistical analysis

Treatment and disease related parameters that were recorded and included in the statistical analysis as predictors of adverse reactions were concurrent chemotherapy, anatomical site of disease, therapy type (definitive or postoperative), type of neck irradiation (bilateral or other) and mean dose to the constrictor muscles of the pharynx (constrictors mean). Primaries arising from the oral cavity and oropharynx were grouped together in a new variable (upper aerodigestive tract malignancies, UATM). Those factors were chosen based on their clinical significance, since the calculated sample size did not allow for more explanatory variables to be included the regression model.

Additional data that was recorded included patient demographics (age, sex) disease stage and treatment parameters regarding dose distribution to the primary site and the neck. More specifically, we calculated the volume in the primary site (PRvol) and lymph node areas (Nvol) that received dose in the high spectrum (≥ 60 Gy) with the intent to compare values among groups.

Descriptive statistics for patient demographics and treatment or disease characteristics included means with standard deviation or medians with interquartile range according to normality assumption for continuous variables, while counts and percentages were presented for categorical variables. Normality assumption was tested by Shapiro-Wilk test. The outcomes of interest were both analyzed as binary variables (dysphagia grade≥ 2 vs grade<2 and PEG no vs PEG yes) and univariate and multivariable logistic regression models were used to investigate for factors associated with outcomes. The factors that were included in the models were therapy type, anatomical site, chemotherapy, type of neck irradiation and mean dose to the constrictors. The mean dose to the constrictors was dichotomized with the intent to detect a cutoff for dysphagia prediction and additional regression analysis was performed to test for associations. Moreover, Mann-Whitney U test was used to compare the median volume of the primary site and neck that received dose in the high spectrum (≥60 Gy) between those patients that developed dysphagia grade ≥2 or needed PEG and those that did not. Multicollinearity of the multivariable models was assessed using the Variance Inflation Factor (VIF) with a threshold value of 4. The odds ratios for each predictor variable in the final model along with their 95% CIs and p-values were presented. For all statistical tests, the significance level was set to a = 0.05.

## Results

Cohort baseline characteristics as well as treatment and disease details are summarized in table 1. 123 participants were included in the study from which 98 were males and 25 females. The patients were treated between January of 2018 and March of 2020 and the mean treatment duration was 6,5 weeks. Average age was 65.95 (SD = 9.97) while 19 suffered from stage I disease, 32 from stage II, 22 from stage III and 50 from stage IV. 38.21% of the patients received RT and 61.79% were submitted to concurrent chemo-RT. Larynx was the most common primary site of disease (41.46%) followed by oropharynx (21.14%), nasopharynx (15.45%), oral cavity (10.57%) and occult primary malignancies (11.38%). Definitive treatment was offered to 83 patients (67.47%) and 40 patients (32.53%) were treated postoperatively. Among the postoperative group, 11 patients were female, average age was 66.12 years, 23 patients had stage IV disease, 6 stage III, 7 stage II and 4 stage I, while 11 presented with a primary in larynx, 25 in the UATM and 4 with occult primary malignancy. Concerning dose prescription, 52.03% were treated to 70 Gy, 45.53% to 60-66 Gy and 2.44% <60 Gy. 73 (59.3%) had positive lymph nodes, while 106 patients (86.17%) received treatment to the primary and neck LN (neck patients) vs 17 (13.82%) that received RT only to the primary site of disease (primary only patients). On the primary site, the median volume that receiving ≥60 Gy was 74.3 cc (IQR = 27.9 cc), 230.9 cc (IQR = 49.4 cc), 82.3 cc (IQR = 169.6 cc), 103.7 cc (IQR = 85.4 cc) for patients with primaries in the larynx, nasopharynx, occult primary and upper aerodigestive tract malignancies respectively, while the corresponding value for the neck was 126.95 cc (IQR = 175.15 cc). Table 2 presents comparisons of dysphagia frequencies among different clinical parameters.

Table 3 summarizes the results of the univariate and multivariable logistic regression models for each possible predictor of dysphagia grade≥ 2. 62 patients developed DP grade≥ 2. In univariate analysis, chemotherapy (p<0.001), constrictors mean (p= 0.002), therapy type (p= 0.042), anatomical site (p= 0.032) and type of neck irradiation (p<0.001) were significantly associated with dysphagia. In multivariable analysis, mean dose to the constrictors, chemotherapy and anatomical site retained significance. More specifically, patients who were treated with radiation in the upper aerodigestive tract were more likely to develop dysphagia (OR = 3.27, 95% CI [1.03 - 10.33], p=0.044) as were those that received chemotherapy (OR = 3.78, 95% CI [1.29 - 11.05], p = .015) or had higher constrictors mean dose (OR = 1.08, 95% CI [1.03 - 1.14], p =.002). Since constrictors mean was significantly associated with dysphagia, we sought to identify a clinically meaningful cut-off that could predict dysphagia manifestation by dichotomizing patients according to that value. The univariate model of constrictors mean dose (as a continuous variable) and dysphagia manifestation was used to estimate a probability of 50% to develop dysphagia and a cut-off at 43 Gy was identified. Then, the new binary variable (<43 Gy vs ≥ 43 Gy) was included in a multivariable model to test its association with dysphagia by adjusting for the rest of the confounders table S1. Constrictors mean value ≥ 43 Gy was significantly associated to dysphagia manifestation (OR = 4.51, 95% CI [1.74 - 11.65], p = .002).

Regarding the secondary outcome, all patients that finally needed PEG (n= 24) belonged to the group that received treatment to the primary site and neck lymph nodes. Table 4 presents the effect of each predictor on the gastrostomy outcome for both univariate and multivariable models. Therapy type (p= 0.026) and anatomical site (p value 0.028 for upper aerodigestive tract and <0.001 for nasopharynx) were found to be significantly associated with PEG use in univariate analysis. The multivariable analysis showed that patients who received radical treatment were more likely to need gastrostomy compared to patients treated postoperatively (OR = 7.03, 95% CI [1.33 - 37.28], p =.022), as were those who were treated in the nasopharynx (OR = 12.62, 95% CI [2.43 - 65.41], p = .003), upper aerodigestive tract (OR = 9.12, 9 5% CI [1.8 - 46.08], p =.007), or had occult primary malignancies (OR= 10.78, 95% CI [1.56, 74.42], p= 0.016).

We then performed Mann-Whitney U test for the whole cohort to compare the median irradiated volume in the neck (Nvol) that received dose ≥ 60 Gy between those that developed DE grade ≥2 or needed PEG and those that did not. Moreover, we performed a subgroup analysis, were we compared the corresponding volumes in the primary site among patients with the same type of cancer. Analysis was possible only for UATM or laryngeal primaries for the primary outcome and for UATM for the secondary due to limited number of patients and observed events in the other cases. Regarding Nvol, patients that developed DP grade ≥2 had a median value of 153.5 cc (IQR=222.5 cc) vs 107.7 cc (IQR= 108.7 cc) for those that did not, with no statistically significant difference observed (p=0.08). The corresponding values regarding the need for PEG use demonstrated a significant difference with higher neck volumes treated to the high spectrum dose in those patients that ultimately needed PEG (240.35 cc ([IQR=180.23 cc] vs 108.3 cc [IQR= 132.15 cc], p=0.015). After comparing the treated volumes in the primary site, those patients with UATM or larynx primaries that developed DP grade ≥2 had median irradiated volume of 147.55 cc (IQR= 83 cc) vs 74.6 cc (IQR= 44.2 cc) for those that did not (p=0.012) and 79.15 cc (IQR=31.3 cc) vs 65.8 cc (IQR= 29.3 cc) respectively (p=0.108). Among patients with upper aerodigestive tract malignancies, those that ultimately needed PEG had a median treated volume of 150.3 cc (IQR= 158.8 cc) vs 93.2 cc (IQR= 84.4 cc) with the difference not being statistically significant (p=0.241).

## Discussion

Adequate control of side effects and subsequent malnutrition during radiation therapy treatment in HNC is significantly important, since there is high level of evidence supporting that excessive weight loss is a negative prognostic factor^4^-^9^. It is believed that this is mainly due to significant weakening of the organism and the immune system as well as deterioration of the patient's general health status. This is turn could result in complications, big treatment breaks or delays^8^,^9^,^11^,^12^. It is well established that therapy interruptions allow for repopulation of cancer cells and increased tumor radioresistance, while breaks of 1 week or greater have been associated with worse prognosis^9^. Moreover, excessive weight loss results in inability to tolerate optimal cancer therapies frequently leading to treatment deintensification and Increased rates of chemotherapy toxicity^7^,^13^,^14^, while postoperative complications have been observed in sarcopenic cancer patients, including HNC^15^,^16^.

In this study we investigated factors associated with dysphagia and need for PEG usage in HNC patients receiving radiation therapy. Constrictor muscles of the pharynx is the most important healthy organ that radiation oncologists pursue to protect during treatment course. CM represents the central swallowing route involving structures of the oropharynx and hypopharynx and RT induced inflammation at that area has been associated with dysphagia, weight loss and treatment interruptions. The most widely used constraint for CM is mean dose ≤ 50 Gy^17^,^18^. In our study we identified an additional, lower cut-off at 43 Gy. According to that novel finding, keeping the mean dose to the CM below 43 Gy whenever possible could result in even lower toxicity rates.

Upper aerodigestive tract malignancies, was the only predictive variable significantly associated with both outcomes of interest. The worse toxicity profile documented in this subgroup of patients is an observation not widely described in the literature. Two retrospective studies^19^,^20^ investigated factors predicting toxicity and weight loss in HNC patients submitted to radiation therapy and found that oral cavity primaries were among the significant predictors. Additionally, we observed that patients with UATM primaries who developed DP grade ≥2 had higher volumes in the primary site of disease treated to dose ≥60 Gy. Treating large volumes, especially in UATM, is common due to prophylactic irradiation for microscopic spread or direct invasion of tumor. This frequently leads to incorporation of more structures involved in swallowing significantly affecting dysphagia. Similar observations have been made in a previous study^21^ of 167 patients with oropharyngeal cancer receiving radiotherapy with IMRT technique. The authors found that grade ≥3 mucositis was associated with the volume of the oral cavity receiving 10.1 Gy and the dose delivered to 21 cc of the oral cavity. Our finding could trigger closer surveillance and nutritional support for those patients treated for UATM cancers especially in the definitive setting (where higher doses and larger RT fields are frequently used) and when concurrent chemotherapy is planned.

PEG represents a more severe condition compared to grade 2 or higher dysphagia, since need for PEG denotes nutritional deficiency that could harm the patient or lead to treatment interruptions. The predictors for PEG usage found in our study were all associated to treatment of the neck lymph nodes. First, all patients that finally needed PEG, received RT to the primary site and neck lymph nodes. Additionally, PEG usage was higher for nasopharyngeal and occult primaries that are traditionally treated with larger neck fields, while Nvol ≥60 Gy was higher in those needing nutritional support by PEG. In our study we did not record the location of pathologic lymph nodes that received higher doses. Nevertheless, toxicity could be related not only to the extent of RT fields but to the location of the lymph nodes treated to higher doses as well. Treating aggressively LN areas that are in close proximity to the swallowing path, could lead to higher adverse events. This is an observation that has not been widely investigated. To our knowledge there is only one recent publication verifying this hypothesis, which found that the toxicity profile in HNC patients is associated with the topography and extent of the disease in the neck stations^22^. Our finding could promote further investigation to identify patients at higher risk of adverse reactions based on the pattern of disease spread in the lymph node stations.

The limitations of our study are first its retrospective design. The results could have been affected by factors such as unbalanced characteristics between participants groups, confounding, selection, recall or misclassification bias. Another limitation of our study is the small sample size. Analysis in a larger sample might had detected associations between variables that were not found in our study but are reasonable according to the literature (e.g. association of concurrent chemotherapy and use of PEG). Moreover, we calculated the number of participants with the intent to detect the effect of adding chemotherapy to RT on the outcomes of interest by adjusting for a specific number of covariates. The logistic model that was produced could not fit more explanatory variables because the study would not have the power to detect their effect on the outcomes. That way we had to exclude variables that would be interesting to investigate and study their impact on the outcomes of interest, such as the effect of high dose distribution, type of chemotherapy or age.

Based on our findings, patients that are going to be treated with radiotherapy for head and neck cancer and are programmed to receive concurrent chemotherapy, have cancer in the oral cavity or oropharynx, or have a calculated mean dose to the constrictors that inevitably exceeds 43 Gy, are at high risk of dysphagia manifestation and close surveillance during treatment should be implemented. This includes control of pain with onset, nutritional support and early intervention for PEG placement when deemed necessary. In those cases, where severe toxicity is expected (definitive treatment of the nasopharynx, oral cavity, oropharynx or OPM especially when concurrent chemotherapy is planned) prophylactic PEG could be considered.

## Supplementary Material

Supplementary table.

## Figures and Tables

**Table 1:** Population baseline characteristics

Baseline characteristics	*n*	*%*
Gender		
Female	25	20.33
Male	98	79.67
Age		
Mean	65.95	
Standard deviation	9.97	
Stage		
Stage I	19	15.44
Stage II	32	26.01
Stage III	22	17.88
Stage IV	50	40.65
Therapy type		
Postoperative	40	32.5
Radical	83	67.5
Anatomical site		
Larynx	51	41.46
Nasopharynx	19	15.45
UATM	39	31.71
OPM	14	11.38
Chemotherapy		
No	47	38.21
Concurrent	76	61.79
Neck		
Other	28	22.76
Bilateral	95	77.24
Toxicity		
Non-severe	61	49.59
Severe	62	50.41
Constrictors mean		
Median	43.91	
IQR	12.38	
PR volume > 60 Gy		
Median	86.90	
IQR	100.55	
Neck volume > 60 Gy		
Median	126.95	
IQR	175.15	

PR volume: the volume in the primary site of disease receiving dose> 60 Gy, neck volume: the volume in the neck receiving dose> 60 Gy, IQR: interquartile range, OPM: occult primary malignancies, UATM: upper aerodigestive tract malignancies

**Table 2:** Frequencies of dysphagia among different clinic parameters

Baseline characteristics	Toxicity 1	Toxicity 2	p-value
Gender			
Female	12 (48%)	13 (52%)	
Male	49 (50%)	49 (50%)	P = 0.999
Stage			
Stage I	11 (57.9%)	8 (42.1%)	
Stage II	21 (65.6%)	11 (34.4%)	
Stage III	7 (31.8%)	15 (68.2%)	
Stage IV	22 (44%)	28 (56%)	P = 0.065
Therapy type			
Postoperative	25 (62.5%)	15 (37.5%)	
Radical	35 (42.7%)	47 (57.3%)	P = 0.062
Anatomical site			
Larynx	29 (56.9%)	22 (43.1%)	
Nasopharynx	5 (26.3%)	14 (73.7%)	
UATM	17 (43.6%)	22 (56.4%)	
OPM	10 (71.4%)	4 (28.6%)	P = 0.03793
Chemotherapy			
No	36 (76.6%)	11 (23.4%)	
Concurrent	25 (32.3%)	51 (67.7%)	P < 0.001
Neck			
Other	23 (82.1%)	5 (17.9%)	
Bilateral	38 (40%)	57 (60%)	P < 0.001
Constrictor mean dose			
Median	42	48.36	P = 0.001
IQR	12.43	10.24	
PR volume > 60 Gy			
Median	74.6	133.8	P = 0.001
IQR	47.7	139.13	
Neck volume > 60 Gy			
Median	68.5	142.8	P = 0.001
IQR	127.45	222.45	
Primary maximum dose			
50 - 54.12 Gy	2 (66.7%)	1 (33.3%)	
60 - 66 Gy	34 (60.7%)	22 (39.3%)	
> 66 Gy	25 (39.1%)	39 (60.9%)	P = 0.028
Neck maximum dose			
No neck RT	14 (82.3%)	3 (17.7%)	
50 - 54.12 Gy	19 (57.6%)	14 (42.4%)	
60 - 66 Gy	19 (40.4%)	28 (59.6%)	
> 66 Gy	9 (36%)	17 (64%)	P = 0.007

Chi square test was used for comparisons. PR volume: the volume in the primary site of disease receiving dose> 60 Gy, neck volume: the volume in the neck receiving dose> 60 Gy, IQR: interquartile range, OPM: occult primary malignancies, UATM: upper aerodigestive tract malignancies

**Table 3:** Univariate and multivariable logistic regression analysis for the association of predictive factors and dysphagia grade ≥ 2

	Univariate analysis		Multivariable analysis	
Predictors	OR (95% C.I.)	p	OR (95% C.I.)	p
Therapy type				
Postoperative				
Radical	2.24 (1.03, 4.86)	0.042*	1.19 (0.41, 3.42)	0.745
Anatomical site				
Larynx				
Nasopharynx	3.56 (1.11, 11.41)	0.032*	2.05 (0.57, 7.37)	0.269
Upper digestive tract	1.65 (0.71, 3.83)	0.247	3.27 (1.03, 10.33)	0.044*
OPM	0.51 (0.14, 1.84)	0.304	1.66 (0.24, 11.47)	0.605
Chemotherapy				
No				
Concurrent	6.49 (2.83, 14.88)	< 0.001*	3.78 (1.29, 11.05)	0.015*
Neck				
Other				
Bilateral	6.6 (2.3, 18.94)	< 0.001*	2.24 (0.62, 8.11)	0.218
Constrictors mean	1.06 (1.02, 1.11)	0.002*	1.08 (1.03, 1.14)	0.002*

OPM: occult primary malignancies

**Table 4:** Univariate and multivariable logistic regression analysis for the association of predictive factors and need for PEG use

	Univariate analysis		Multivariable analysis	
Predictors	OR (95% C.I.)	*p*	OR (95% C.I.)	*p*
Therapy type				
Postoperative				
Radical	4.25 (1.18, 15.22)	0.026*	7.03 (1.33, 37.28)	0.022*
Anatomical site				
Larynx				
Nasopharynx	14.1 (3.23, 61.58)	< 0.001*	12.62 (2.43, 65.41)	0.003*
Upper digestive tract	4.7 (1.18, 18.77)	0.028	9.12 (1.8, 46.08)	0.007*
OPM	4.27 (0.76, 24.09)	0.1	10.78 (1.56, 74.42)	0.016*
Chemotherapy				
No				
Concurrent	2.73 (0.94, 7.92)	0.064	0.6 (0.12, 2.95)	0.527
Neck bilateral				
Other				
Bilateral	3.77 (0.83, 17.17)	0.087	2.18 (0.3, 15.83)	0.442
Constrictors mean	1 (0.9591, 1.0476)	0.916	0.99 (0.9406, 1.0557)	0.905

OPM: occult primary malignancies
