# Supplementary material for: Investigating factors associated to dysphagia and need for percutaneous endoscopic gastrostomy in patients with head and neck cancer receiving radiation therapy
Source: J Cancer. 2022 Feb 28;13(5):1523–9. doi: 10.7150/jca.69130 (PMC8965122; doi:10.7150/jca.69130)
Supplement: Supplementary file 1 — Supplementary table. [file jcav13p1523s1.pdf]

Table S1

Univariate and multivariable logistic regression analysis for the association of predictive factors and dysphagia grade≥ 2 (constrictors as a categorical variable)				
	Univariate analysis		Multivariable analysis	
Predictors	OR (95% C.I.)	*p*	OR (95% C.I.)	*p*
Therapy type				
Postoperative				
Radical	2.24 (1.03, 4.86)	0.042*	1.31 (0.46, 3.7)	0.616
Anatomical site				
Larynx				
Nasopharynx	3.56 (1.11, 11.41)	0.032*	2.8 (0.71, 11.04)	0.141
Upper digestive tract	1.65 (0.71, 3.83)	0.247	3.22 (1.02, 10.18)	0.047*
OPM	0.51 (0.14, 1.84)	0.304	1.47 (0.25, 8.57)	0.671
Chemotherapy				
No				
Concurrent	6.49 (2.83, 14.88)	< 0.001*	3.05 (1.06, 8.81)	0.039*
Neck				
Other				
Bilateral	6.6 (2.3, 18.94)	< 0.001*	2.5 (0.71, 8.84)	0.153
Constrictors group	3.38 (1.6, 7.11)	0.001*	4.51 (1.74, 11.65)	0.002*
OPM: occult primary malignancies				
